# Machine learning-based gait adaptation dysfunction identification using CMill-based gait data

**DOI:** 10.3389/fnbot.2024.1421401

**Published:** 2024-07-29

**Authors:** Hang Yang, Zhenyi Liao, Hailei Zou, Kuncheng Li, Ye Zhou, Zhenzhen Gao, Yajun Mao, Caiping Song

**Affiliations:** ^1^Department of Rehabilitation Medicine, the First Affiliated Hospital of Zhejiang Chinese Medical University, Zhejiang, China; ^2^College of Science, China Jiliang University, Zhejiang, China; ^3^MeritData Technology Co., Ltd., Shanxi, China

**Keywords:** gait adaptability, stroke rehabilitation, machine learning, AdaCost algorithm, diagnostic model

## Abstract

**Background:**

Combining machine learning (ML) with gait analysis is widely applicable for diagnosing abnormal gait patterns.

**Objective:**

To analyze gait adaptability characteristics in stroke patients, develop ML models to identify individuals with GAD, and select optimal diagnostic models and key classification features.

**Methods:**

This study was investigated with 30 stroke patients (mean age 42.69 years, 60% male) and 50 healthy adults (mean age 41.34 years, 58% male). Gait adaptability was assessed using a CMill treadmill on gait adaptation tasks: target stepping, slalom walking, obstacle avoidance, and speed adaptation. The preliminary analysis of variables in both groups was conducted using t-tests and Pearson correlation. Features were extracted from demographics, gait kinematics, and gait adaptability datasets. ML models based on Support Vector Machine, Decision Tree, Multi-layer Perceptron, K-Nearest Neighbors, and AdaCost algorithm were trained to classify individuals with and without GAD. Model performance was evaluated using accuracy (ACC), sensitivity (SEN), F1-score and the area under the receiver operating characteristic (ROC) curve (AUC).

**Results:**

The stroke group showed a significantly decreased gait speed (*p* = 0.000) and step length (SL) (*p* = 0.000), while the asymmetry of SL (*p* = 0.000) and ST (*p* = 0.000) was higher compared to the healthy group. The gait adaptation tasks significantly decreased in slalom walking (*p* = 0.000), obstacle avoidance (*p* = 0.000), and speed adaptation (*p* = 0.000). Gait speed (*p* = 0.000) and obstacle avoidance (*p* = 0.000) were significantly correlated with global F-A score in stroke patients. The AdaCost demonstrated better classification performance with an ACC of 0.85, SEN of 0.80, F1-score of 0.77, and ROC-AUC of 0.75. Obstacle avoidance and gait speed were identified as critical features in this model.

**Conclusion:**

Stroke patients walk slower with shorter SL and more asymmetry of SL and ST. Their gait adaptability was decreased, particularly in obstacle avoidance and speed adaptation. The faster gait speed and better obstacle avoidance were correlated with better functional mobility. The AdaCost identifies individuals with GAD and facilitates clinical decision-making. This advances the future development of user-friendly interfaces and computer-aided diagnosis systems.

## Introduction

Stroke is one of the foremost causes of disability among patients (Renedo et al., 2024). Approximately 70 to 80% of stroke survivors are discharged with persistent impairments in gait speed and walking distance, failing to meet the criteria for community ambulation, thereby increasing the risk of falls (Zhong et al., 2021). Adaptive walking is a prerequisite for safe community ambulation, requiring patients to adjust their gaits to environment, navigate turns and obstacles and form a tripartite model of locomotor control characterized by stepping and stability (Hollands et al., 2013; Balasubramanian et al., 2014). Early evaluation and diagnosis of gait adaptation dysfunction (GAD) is critical for rehabilitation effect in stroke patients.

The assessment of gait adaptability currently receives limited clinical attention. Some simple walking function tests (e.g., 10-meter walk test, timed up and go test) are commonly used to evaluate and predict walking recovery (Yang et al., 2024). However, most gait adaptability outcomes show little or only moderate correlation with clinical walking and balance (Geerse et al., 2021). Considering the complex nature of the construct of gait adaptability, Balasubramanian et al. (2014) developed a comprehensive assessment encompassing nine domains, including obstacle negotiation, timing, environment, cognition, etc. Despite its thoroughness, the validity, reliability, and specificity of this “observational gait analysis” remain uncertain (Ferrarello et al., 2013). Instrumented gait analysis, using pressure sensors or motion capture systems with or without markers, provides quantitative kinematic and kinetic parameters (Nadeau et al., 2013). It is widely regarded as the gold standard for gait analysis. However, the measurement is laborious and requires trained personnel, involving large datasets and complex computations (Broström et al., 2012), making it rarely used for definitive clinical diagnosis (Baker et al., 2016) and challenging to identify specific domains of GAD. Additionally, most clinical studies evaluate only one domain of gait adaptability constructs, primarily obstacle negotiation (Van Swigchem et al., 2014; Van Ooijen et al., 2015). The augmented reality (AR)-based CMill treadmill can design environments for gait adaptability, enabling real-time recording of kinematic and kinetic gait data. Studies have demonstrated the validity and reproducibility of the CMill as a measurement tool (Tuijtelaars et al., 2021). Additionally, another study employed success rates of CMill gait adaptation task to develop a model for detecting freezing of gait (FOG) in Parkinson’s disease (PD) using stepwise discriminant analysis (Chen et al., 2021).

Machine learning (ML) combined with gait analysis has been extensively applied in fall detection, human pose tracking, and person identification and authentication. Among the most prevalent applications are disease diagnosis and monitoring (Harris et al., 2022). In the field of neurorehabilitation, many ML models have been developed to identify pathological gait (Ye et al., 2018), supporting clinical rehabilitation decision-making. Lau et al. (2009) used two portable sensors to collect kinematic data from seven stroke patients with foot drop. They employed a support vector machine (SVM) algorithm, which identified five different walking patterns with an accuracy of 97.5%. Cui et al. (2018) used sensors and electromyography (EMG) to collect trajectory markers, kinematics, and EMG signals from participants during walking. They trained seven commonly used decision fusion algorithms (such as SVM, random forest (RF), artificial neural network) using different data modalities to distinguish individuals with hemiparetic gait. These biomechanical data measured in gait laboratory settings are inefficient for clinical practice. Yet, traditional gait capability tests still retain predictive capabilities. Abdollahi et al. (2024) utilized common clinical tests for stroke patients (balance, 10-meter walk test, timed up-and-go (TUG)) alongside kinematic data. They employed supervised learning algorithms (SVM, RF, and logistic regression) to differentiate between high and low fall risk, with RF achieving the highest accuracy and balance and TUG tests being most effective predictors of falls. However, there is currently no ML model utilizing CMill gait features for classifying GAD.

Classification is the most widely applied ML task in the medical field (Yin et al., 2013). Traditional ML often assumes a relatively balanced class distribution, but class imbalance is common in clinical settings (Bak and Jensen, 2016). Recently, adaptive boosting (AdaBoost) has emerged as a leading ensemble learning technique to address class imbalance, widely used in healthcare (Hatwell et al., 2020; Lu et al., 2021). Diagnostic problems often involve high costs of misclassification, especially misdiagnosis. Proper misclassification cost considerations are crucial for ML performance. The adaptive cost-sensitive algorithm (AdaCost) incorporates a cost function into the AdaBoost training sequence, emphasizing the classification of minority classes and improving overall performance. The AdaCost algorithm has been used to improve prediction accuracy in various fields, including transformer fault analysis (Hechifa et al., 2024), tunnel excavation rock mass prediction (Mengqi et al., 2020), lung nodule diagnosis (Jinzhu et al., 2009), and high-risk human papillomavirus diagnosis (Park et al., 2003). To address the resource-constrained settings with small datasets, Fraiwan and Hassanin (2021) found the combination of vertical ground reaction force-based features and the Adaboost classification model had the highest classification accuracy in detecting degenerative neuromuscular disease. However, studies using AdaCost as the primary ensemble learning method for gait classification remain limited.

AR-based CMill treadmill gait adaptation training is promising. However, the lack of comprehensive and standardized walking-adaptability testing (Balasubramanian et al., 2014), combined with insufficient attention to the decline in patients’ gait adaptability, leads to inadequate recognition and diagnosis of this functional impairment, thereby limiting the effectiveness of post-stroke rehabilitation. Developing efficient ML models based on clinical measurements to aid in diagnosing GAD and optimizing rehabilitation programs remains a crucial issue.

This study aims to simplify data measurement by using demographics, gait kinematics, and gait adaptability features to train ML models. These models will identify individuals with post-stroke GAD, aiding clinical diagnosis and the development of personalized rehabilitation plans.

## Methods

### Subjects

This study enrolled 30 stroke patients from the Rehabilitation Department of the First Affiliated Hospital of Zhejiang Medical University, along with 50 healthy volunteers. This study was approved by the Ethics Committee of the First Affiliated Hospital of Zhejiang Chinese Medical University (Ethics Number: [2021-KL-187-02]), and informed consent was obtained from all participants.

The inclusion criteria for stroke rehabilitation patients were as follows: (1) first-time stroke resulting in unilateral hemiparesis confirmed by CT or MRI, with a duration of less than 180 days; (2) age between 20 and 65 years old; (3) adequate communication skills to follow instructions; (4) ability to walk at least 10 meters under indoor supervision or Functional Ambulation Category (FAC) score of two.

Inclusion criteria for healthy participants were as follows: (1) aged between 20 and 65 years old; (2) absence of known musculoskeletal, neurological, cardiovascular, or other conditions affecting walking ability; (3) normal cognitive function and following instructions.

Exclusion criteria for both groups were: (1) body weight ≥ 135 kg, height ≥ two meters; (2) severe visual or auditory impairments affecting walking; (3) severe neurological or lower limb disorders, respiratory or cardiovascular diseases, psychiatric disorders, and pregnancy that affect the walking ability and gait patterns.

### Measurement

#### CMill treadmill

Participants were assessed using the CMill VR+ treadmill (Motek Medical B.V, Netherlands) (Supplementary Figure S1), which employs AR to project virtual objects onto the treadmill, providing visual and auditory cues. The treadmill features an embedded force plate recording gait parameters at 500 Hz. Participants completed one to two practice sessions for familiarization. Stroke participants wore safety harnesses per standard protocol during testing, supervised by a rehabilitation physician and a physical therapist.

#### The program of CMill gait adaptation task

**Figure 1:**
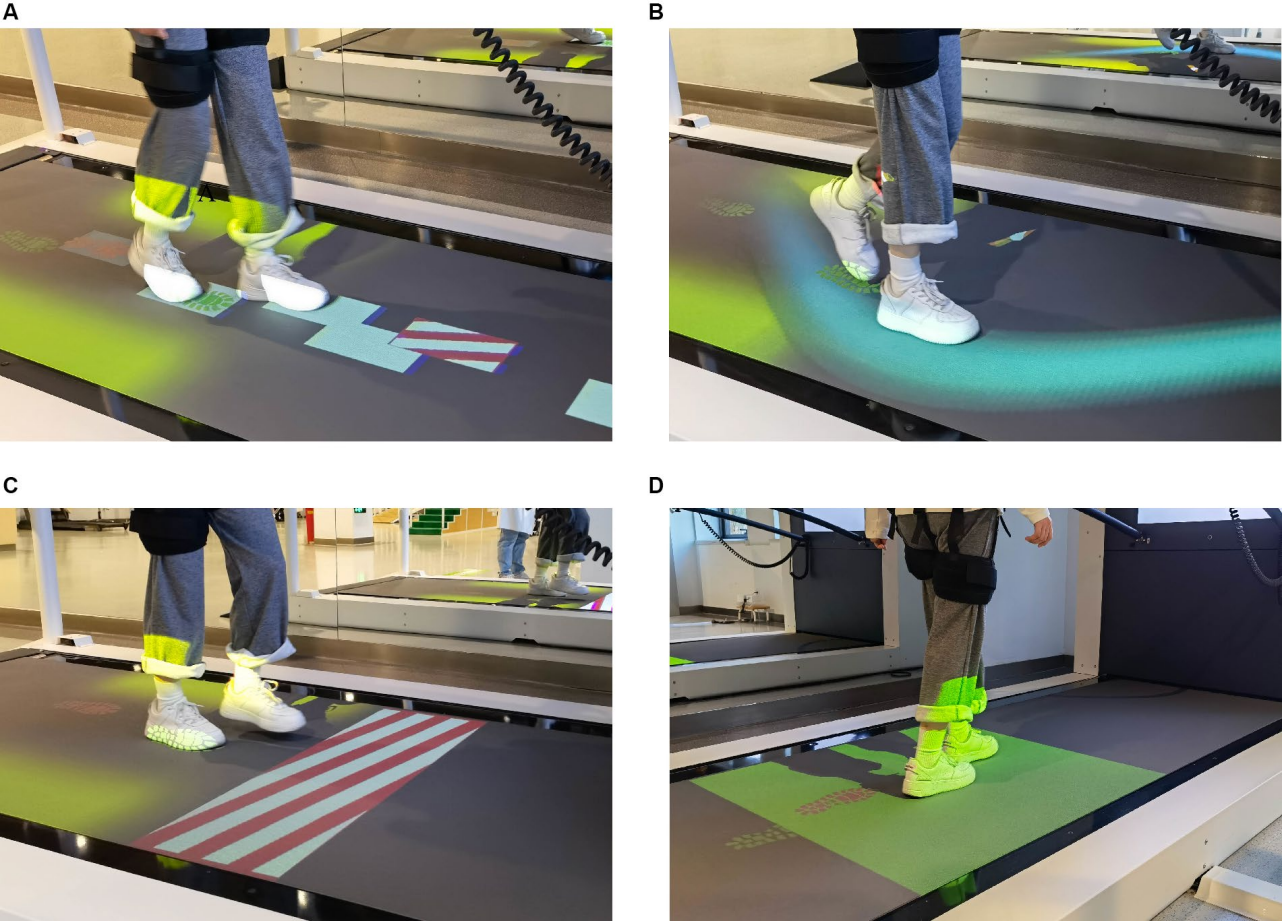
The gait adaptation task. **(A)** Target stepping. Participants were instructed to step on random targets with visual guidance. **(B)** Slalom walking. Participants keep their feet walking in the virtual curve of the treadmill. **(C)** Obstacle avoidance. Participants need to cross or avoid the obstacles with both feet. **(D)** Speed adaptation. Participants adjust their gait and speed to keep their feet always walking in the target area.

The CMill gait adaptation program includes a warm-up period and four gait adaptation tasks (as shown in Figure 1 for task scenarios; Table 1 for task details). Before each session, the treadmill’s force plate is reset. Participants warm up at a chosen speed, then sequentially complete the target stepping, slalom walking, obstacle avoidance, and speed adaptation tasks with visual and auditory cues. Each task lasts approximately 2–3 min with 30-s walking intervals, totaling around 15 min.

**Table 1:** The description of CMill gait adaptation program and clinical function measure.

Measurement	Description
CMill gait adaptation program	
Warm-up	Participants walk on CMill treadmill without guidance and select a comfortable speed.
Target stepping	Instruct the participants to step on the virtual targets projected on the belt to practice foot positioning.
Slalom walking	A slalom pathway was generated on the belt. Participants need to navigate the curve to improve motor control
Obstacle avoidance	Participants take steps to avoid visual obstacles appearing intermittently ahead.
Speed adaptation	Participants were instructed to keep walking within the changing target area, either accelerating or decelerating to adjust their gait
Clinical function measures	
FAC (level)	Level 0: inability to stand and walk; Level 1: indoor ambulation with assistance within 10 meters; Level 2: indoor ambulation of up to 20 meters under supervision; Level 3: independent indoor ambulation of 50 meters; Level 4: continuous walking of over 100 meters and ability to cross typical obstacles; Level 5: independent outdoor ambulation.
ADL (score)	The Modified Barthel Index was used to assess ADL with a maximum score of 100. A score of 60 indicates self-sufficiency, with higher scores indicating better self-care ability and less dependence.

FAC, functional ambulation category; ADL, activities of daily living.

#### Clinical function measures

FAC and Activities of Daily Life (ADL) were used to assess participants’ walking ability and self-care ability, respectively (Table 1). Based on the FAC and ADL, a global F-A score was created by standardizing the FAC and ADL scores and using their average as the overall observation of mobility.

### Data acquisition and preprocessing

Clinical baseline characteristics include demographics and mobility function performance through interviews, while the gait data were automatically collected, calculated, and reported by CMill. The composite dataset includes 18 variables: 8 clinical baseline variables, 6 gait kinematic variables, and 4 gait adaptability variables (success rate determined by CMill based on foot positions relative to virtual targets, obstacles, or pathways). Detailed definitions and explanations for each variable are provided in Supplementary Table S1.

For data preprocessing, outliers were removed based on the principle of being greater or less than three standard deviations. Missing values for different data types were imputed using either the mean or the mode.

### Data analysis

Before applying ML, a preliminary analysis was conducted on the clinical baseline and gait characteristics of the two groups. We employed SPSS 22 to perform t-tests between the two groups. This analysis helped assess whether the variables could be extracted as feature inputs. Pearson correlation coefficient (r) was employed to assess the relationship between different variables and the global F-A score within the stroke group. The sign (positive or negative) of the r indicates the direction of the correlation, with stronger correlations approaching an absolute value of one. A significance level of *p* < 0.05 was used to determine statistically meaningful differences.

### Establishment of machine learning diagnosis models

**Figure 2:**
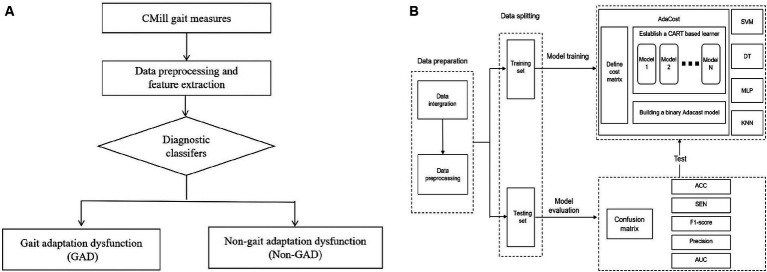
Establishment of machine learning diagnosis model. **(A)** The aided diagnosis basic flowchart. The workflow of the simple diagnostic classifier includes: (1) Participants complete basic assessments. (2) Data is preprocessed, and features are extracted and selected. (3) The diagnostic classifier is applied. (4) The classifier maps gait data to GAD and non-GAD output spaces based on the data features. **(B)** Training and testing of diagnostic classifier. The process outlines the steps of model establishment, including data preparation, data splitting, and model training and testing. The training set is used to train five major classifiers, with AdaCost as an example (cost matrix and base learner selection). The test set evaluates classifier performance, calculating metrics based on the confusion matrix. GAD, gait adaptation dysfunction; CART, classification and regression trees; SVM, support vector machine; DT, decision tree; MLP, multi-layer perceptron; KNN, k-nearest neighbors; Adacost, adaptive cost-sensitive algorithm; ACC, accuracy; SEN, sensitivity; AUC, the area under the receiver operating characteristic curve.

The ML model consists of input, training, testing, and performance evaluation. The objective is to compare the performance of different classifiers to generate the best ML model for classifying individuals as having or not having post-stroke GAD (Figure 2A). Key steps include data preparation, data splitting, model training, and evaluation.

#### Data preparation

Feature Selection: There is no unified standard for feature selection in ML (Bush et al., 1992). We referenced the two groups’ statistical differences and correlation results, along with findings from other ML studies (Rodrigo et al., 2021; Al-Ramini et al., 2022) and correlation analyses (Dommershuijsen et al., 2020; Guzelbulut et al., 2022). Based on these, 14 variables were extracted from the dataset as feature inputs (Table 2), using binary labels of having or not having post-stroke GAD for supervised learning.

**Table 2:** Features extraction as inputs.

Number	Data source	Features
1	A: Clinical baseline characteristics (demographics)	A1: Age (y)
2		A2: Gender (male/female)
3		A3: Height (m)
4		A4: Weight (kg)
5	B: Gait kinematics (gait spatiotemporal parameters)	B1: Gait speed (km/h)
6		B2: Step width (m)
7		B3: SL (m)
8		B4: ST (s)
9		B5: ASL
10		B6: AST
11	C: Gait adaptability (success rate of gait adaptation task)	C1: Target stepping
12		C2: Slalom walking
13		C3: Obstacle avoidance
14		C4: Speed adaptation

SL, step length; ST, stance time; ASL, asymmetry of step length; AST, asymmetry of stance time.

#### Data splitting

The dataset was divided into training and testing sets in an 80:20 ratio using the sklearn library in Python.

#### Model training

Binary classification tasks were constructed using SVM, decision tree (DT), Multi-Layer Perceptron (MLP), K-Nearest Neighbors (KNN), and AdaCost algorithms, resulting in corresponding models. The principles and decision-making processes of each algorithm vary, as shown in Supplementary Table S2. Random search was used for hyperparameter tuning across all models. Given our dataset’s characteristics, we hypothesize that the AdaCost algorithm will achieve superior performance. The training process for this model is as follows. (Figure 2B).

1. Selecting Base Learner: The classification and regression trees (CART) algorithm was chosen as the base learner for the AdaCost. This binary tree recursively builds the classification tree from the root node using the training set.
2. Defining the Cost Matrix: A 2×2 cost matrix *C* was defined, where the element *C*_ij_ represents the misclassification cost of incorrectly diagnosing a normal gait adaptability as impaired. This reflects the relative diagnostic loss due to misclassification of gait adaptability states in the assessment of GAD.
3. Hyperparameter Tuning: It was conducted using a random search strategy with 50 iterations.
4. Training the Model: The ML model was developed in a Python 3.8 environment on a Windows operating system. Relevant libraries were used to set model parameters and match input and output variables.

#### Model evaluation

In binary classification models, performance metrics such as accuracy (ACC), sensitivity (SEN), F1-score, precision, and the area under the receiver operating characteristic (ROC) curve (AUC) can be calculated based on the confusion matrix (Supplementary Tables S3, S4). ACC is the initial assessment that represents the true results, while the F1-score is a single metric in imbalanced datasets. SEN indicates the model’s ability to identify participants with GAD, and precision assess a low false positive rate. ROC-AUC evaluates the classifier’s ability to identify between classes. Higher values of these metrics closer to one, indicate better model performance.

## Results

### Descriptive analysis

Clinical baseline characteristics: The stroke group included 18 males with a mean age of 42.69 years, height of 1.62 m, and weight of 57.9 kg. The healthy group comprised 29 males with a mean age of 41.34 years, height of 1.65 m, and weight of 62.37 kg. There were no significant differences in demographics between the two groups (*p* > 0.05). The average onset of stroke was 99 days, with 6 individuals (20%) experiencing left hemiplegia. The mean level of FAC was 2.2 (*t* = −19.66, *p* = 0.000), and the ADL score was 66 (*t* = −15.86, *p* = 0.000), both significantly lower compared to the healthy group. (Table 3).

**Table 3:** Comparison of the variables between the two groups.

	Healthy group (*n* = 50)	Stroke group (*n* = 30)		*p*-value
Clinical baseline characteristics				
Age, y	41.34 (8.01)	42.69 (6.54)		0.415
Male	29 (58%)	18 (60%)		0.860
Height, m	1.65 (0.06)	1.62 (0.08)		0.082
Weight, kg	62.37 (9.42)	57.9 (13.9)		0.126
Onset of stroke, d	–	99 (55.6)		–
Affected side (left)	–	6 (20%)		–
FAC	5 (0)	2.2 (0.78)		0.000*
ADL	100 (0)	66 (11.74)		0.000*
Gait kinematics (gait spatiotemporal parameters)				
Gait speed, km/h	2.69 (0.47)	1.01 (0.49)		0.000*
Step width, m	0.15 (0.03)	0.16 (0.04)		0.329
		Affected side	Unaffected side	
SL, m	0.44 (0.06)	0.25 (0.09)*	0.20 (0.06)*#	
ST, s	0.40 (0.07)	0.36 (0.17)	0.59 (0.23)*#	
ASL	1.01 (0.03)	1.44 (0.35)		0.000*
AST	1.00 (0.06)	2.19 (0.98)		0.000*
Gait adaptability (success rate of gait adaptation task)				
Target stepping, %	95.93 (4.33)	95.01 (3.59)		0.309
Slalom walking, %	96.92 (3.77)	87.03 (3.90)		0.000*
Obstacle avoidance, %	96.2 (3.67)	73.1 (14.4)		0.000
Speed adaptation, %	97.92 (3.50)	92.60 (4.32)		0.000

Continuous variables are presented as mean (SD); Categorical variables presented as number (%); FAC, functional ambulation category; ADL, activities of daily living; SL, step length; ST, single stance time; ASL, asymmetry of step length; AST, asymmetry of stance time. *The comparison between the two groups, *p* < 0.05. #The comparison between affected side and unaffected side within the stroke group, *P* < 0.05.

Gait kinematics and adaptability: In Table 3, compared to the healthy group, the stroke group exhibited a significantly lower average gait speed of 1.01 km/h (*t* = −15.07, *p* = 0.000), step length (SL) on the affected side of 0.25 m (*t* = −10.27, *p* = 0.000), and SL on the unaffected side of 0.20 m (*t* = −17.32, *p* = 0.000). Stroke patients showed a significantly higher asymmetry of SL (ASL) at 1.44 (*t* = 6.71, *p* = 0.000), and asymmetry of ST (AST) at 2.19 (*t* = 6.64, *p* = 0.000), as well as lower success rates of 87.03% (*t* = −11.12, *p* = 0.000), 73.1% (*t* = −8.62, *p* = 0.000), and 92.6% (*t* = −5.71, *p* = 0.000) for slalom walking, obstacle avoidance, and speed adaptation, respectively. Additionally, differences within the limbs in stroke patients were significant, with SL longer on the affected side at 0.25 m (*t* = −2.53, *p* = 0.01), but ST shorter on the affected side at 0.36 s (*t* = 4.40, *p* = 0.000).

### Pearson correlation analysis

The correlation between various variables and the global F-A score in the stroke group was assessed (Table 4). The onset of stroke (*r* = −0.798, *p* = 0.000) and the affected side (*r* = −0.912, *p* = 0.000) were significantly negatively correlated with the global F-A score. Gait speed (*r* = 0.803, *p* = 0.000) and obstacle avoidance (*r* = 0.823, *p* = 0.000) have strongly positive correlations with the global F-A score.

**Table 4:** The correlation between variables and clinical function measure (global F-A) for stroke group.

	Coefficient (r)	*P*-value
Clinical baseline characteristics		
Age, y	−0.069	0.639
Gender (male-to-female ratio)	0.177	0.256
Height, m	0.167	0.247
Weight, kg	0.173	0.230
Onset of stroke, d	−0.798	0.000
Affected side (left-to-right ratio)	−0.912	0.000
Gait kinematics (gait spatiotemporal parameters)		
Gait speed, km/h	0.803	0.000
Step width, m	−0.100	0.49
SL-affected side, m	−0.266	0.256
SL-unaffected side, m	−0.355	0.125
ASL	−0.250	0.288
ST-affected side, s	0.425	0.062
ST-unaffected side, s	0.441	0.051
AST	−0.003	0.989
Gait adaptability (success rate of gait adaptation task)		
Target stepping, %	0.388	0.091
Slalom walking, %	0.286	0.044
Obstacle avoidance, %	0.823	0.000
Speed adaptation, %	0.202	0.159

SL, step length; ASL, asymmetry of step length; ST, stance time; AST, asymmetry of stance time.

The significant correlation suggests that faster gait speed and better gait adaptability of obstacle avoidance are associated with better functional mobility.

### ML models results

**Figure 3:**
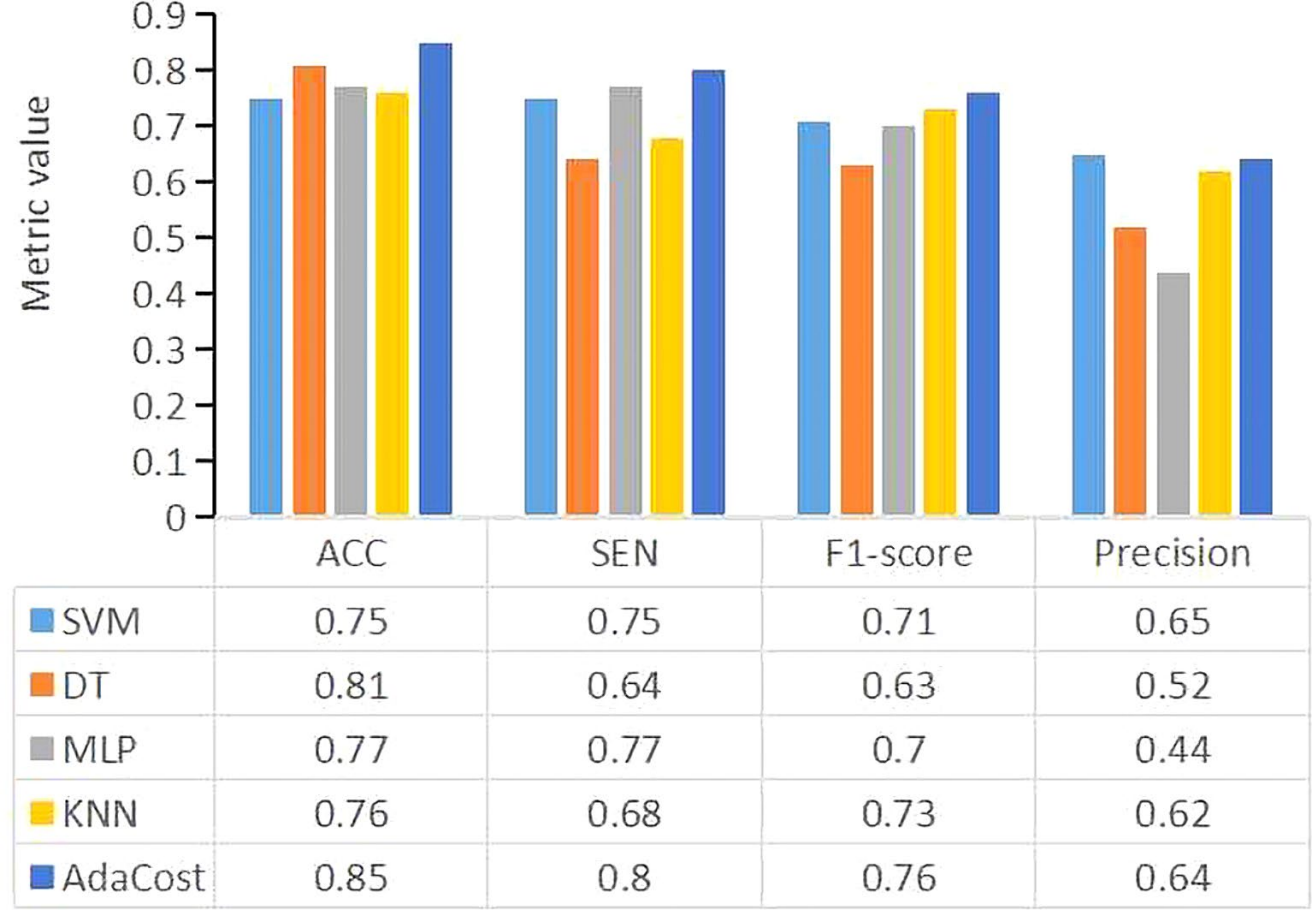
The performance metrics for each machine learning algorithm model. The X-axis represents the evaluation metrics, and the Y-axis represents the metric values. Different colors of the bar represent different classifiers. SVM, support vector machine; DT, decision tree; MLP, multi-layer perceptron; KNN, k-nearest neighbors; AdaCost, adaptive cost-sensitive algorithm; ACC, accuracy; SEN, sensitivity.

In Figure 3, AdaCost shows the highest ACC at 0.85, followed by DT at 0.81, and SVM, MLP, and KNN were below 0.8.

The SEN of AdaCost model is 0.8, followed by MLP (0.77) and SVM (0.75), with DT and KNN scoring below 0.70.

The precision of AdaCost is 0.64, lower than SVM of 0.65 and other ML models.

Using F1-score as a single evaluation metric for imbalanced datasets, AdaCost achieved 0.76, followed by KNN (0.73), SVM (0.71), MLP (0.70) and DT (0.63).

**Figure 4:**
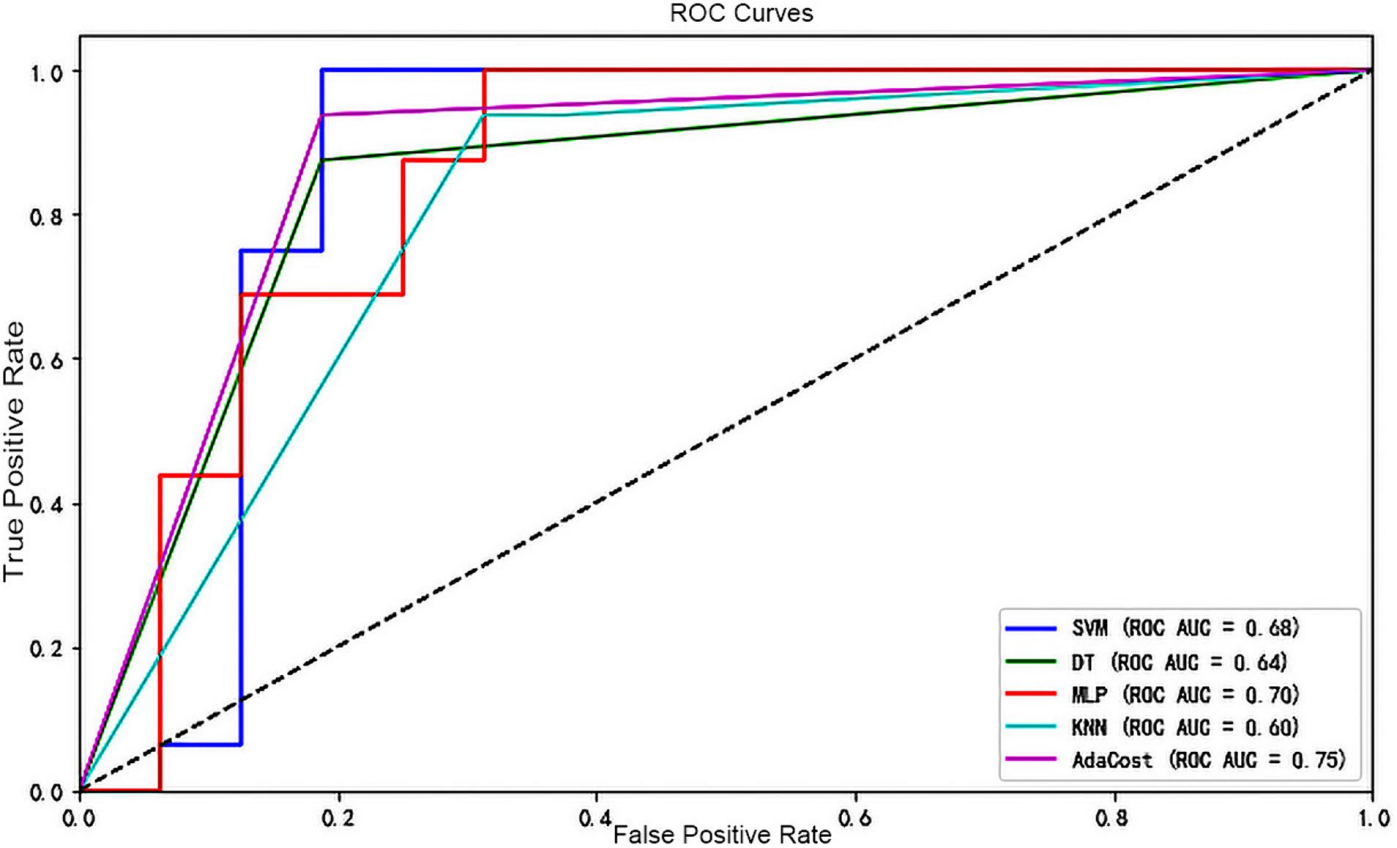
ROC curve of each machine learning algorithm model. The X-axis represents the false positive rate and the Y-axis represents the true positive rate. Different colors of the line represent different classifiers. A dashed line runs from the bottom left corner (0,0) to the top right corner (1,1), indicating the scenario where the false positive rate equals the true positive rate (the random guessing). If a classifier’s ROC curve is above this dashed line, it indicates performance better than random guessing and vice versa. ROC, receiver operating characteristic; AUC, the area under the receiver operating characteristic curve; SVM, support vector machine; DT, decision tree; MLP, multi-layer perceptron; KNN, k-nearest neighbors; AdaCost, adaptive cost-sensitive algorithm.

Lastly, as shown in Figure 4, the ROC-AUC for AdaCost was 0.75, larger than MLP (0.7). The area of SVM, DT and KNN was all below 0.7.

**Figure 5:**
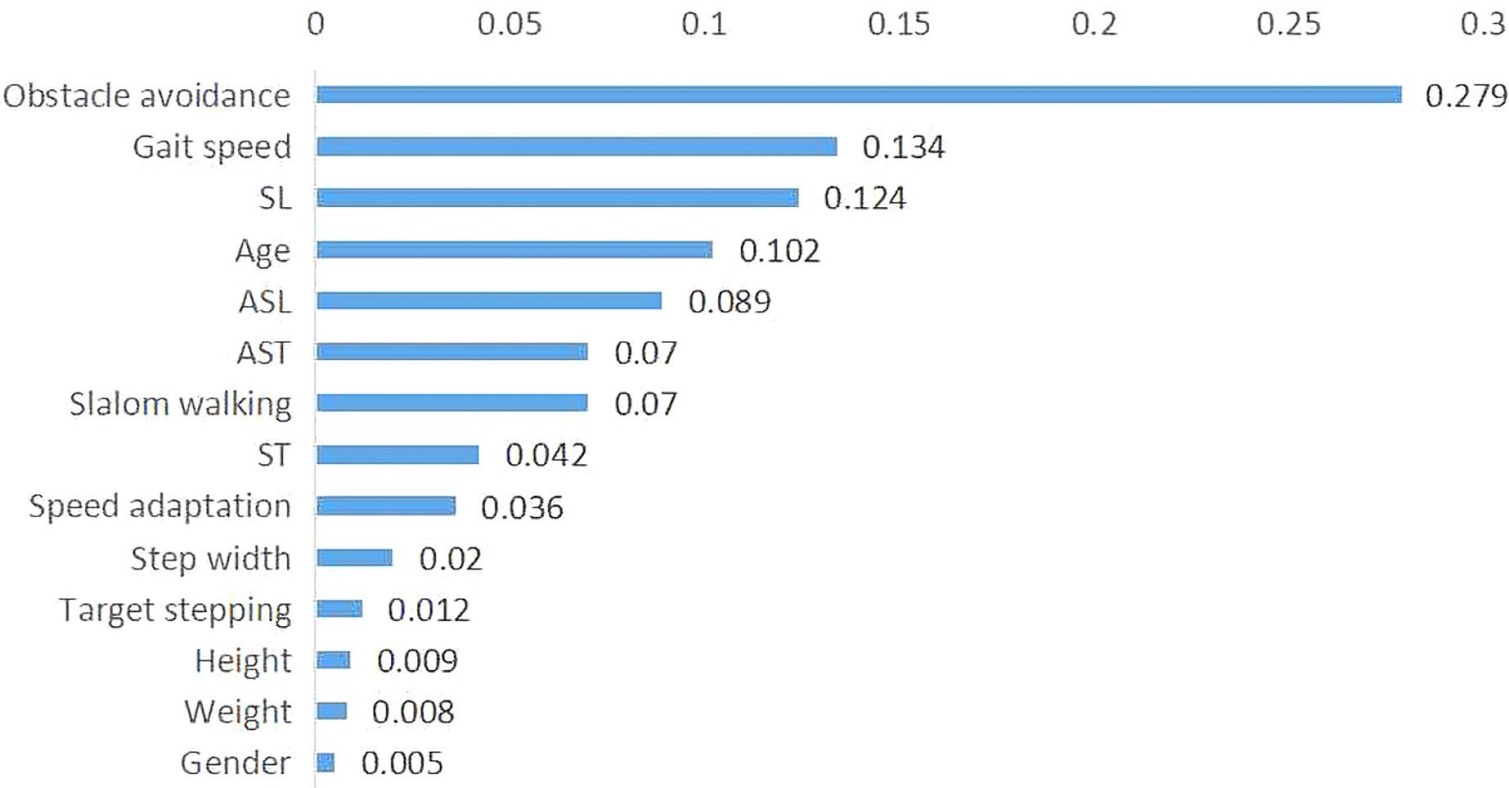
Importance of features in the AdaCost algorithm model. The X-axis represents the feature importance scores, which reflect the contribution of each feature to the model predictions, and the Y-axis represents the feature names or labels. The sum of importance scores for all features is one. AdaCost, adaptive cost-sensitive algorithm; SL, step length; ST, single stance time; ASL, asymmetry of step length; AST, asymmetry of stance time.

Notably, the important classification features were obtained based on the weight of each feature in the AdaCost ensemble learning model, ranging from 0.5 to 27.9% (Figure 5). The top five most important features were obstacle avoidance (27.9%), gait speed (13.4%), SL, age, and ASL. Obstacle avoidance and gait speed were the representatives of gait adaptability and kinematics, corroborating their strong correlation with global F-A scores.

The models’ results indicate AdaCost algorithm provides higher accuracy, sensitivity, and robustness in clinical settings for early identification of GAD.

## Discussion

Based on ML algorithms, using demographics and gait features of 30 stroke patients and 50 healthy controls as inputs, individuals were classified with or without GAD. Results showed that: (1) Stroke patients’ gait kinetics and adaptability decreased in gait speed (*p* = 0.000), SL (*p* = 0.000), obstacle avoidance (*p* = 0.000) and speed adaptation (*p* = 0.000) with more asymmetry of limbs (*p* = 0.000). (2) All ML models could identify individuals with GAD, with the AdaCost achieving the best performance (ACC = 0.83, SEN = 0.8, AUC = 0.75). (3) Obstacle avoidance and gait speed were the critical features for classification in this mode.

GAD is a common dysfunction in stroke patients, but there is no “gold standard” assessment. C-Mill can be used to evaluate gait adaptability (Tuijtelaars et al., 2021, 2022). Tuijtelaars et al. (2021) used the C-Mill to set target stepping and obstacle avoidance tasks, validating the hypothesis that gait adaptability is decreased in polio patients. However, they did not analyze other domains of gait adaptability. Our study found that stroke patients also exhibit reduced abilities in slalom walking and speed adaptation. The gait kinematics assessed by C-Mill are generally consistent with the gait characteristics of elderly hemiplegic patients as reported previously (Sang et al., 2013). However, there are significant differences in the patterns of ASL among different stroke patients (Balasubramanian et al., 2007; Allen et al., 2011). The SL on the affected side is longer in our finding, which is related to the weakened propulsive force of the affected limb, suggesting the impaired dorsiflexion of the affected ankle or an altered gait pattern on the treadmill (Soni and Lamontagne, 2021). The gait assessment validated the feasibility of CMill for gait adaptability evaluation and provided a partial reference for feature selection, addressing gaps in clinical walking adaptability assessments.

Many studies have focused on integrating artificial intelligence (AI) with gait analysis for hemiparetic gait analysis and diagnostic prediction. Li et al. extracted new gait features (dynamic time warping distance, sample entropy, and empirical mode decomposition) to evaluate gait symmetry, regularity, and stability. They used classical algorithms (SVM and KNN) to assess the recognition of these new gait features for hemiparetic gait (Li et al., 2019). Although new features in classification can achieve good performance, these features may lack clear clinical significance. Moreover, many ML models are black boxes, often unable to be explained (Hatwell et al., 2020). We found that superior performance in obstacle avoidance and faster gait speed were positively correlated with improved functional mobility. These two variables, obstacle avoidance and gait speed, were also identified as significant features in the AdaCost model. The consistency between correlation analysis and model feature importance partly interprets the ML clinical significance and can further guide rehabilitation strategies for GAD, highlighting the importance of prioritizing obstacle avoidance and gait speed in rehabilitation programs. The correlation results are consistent with previous findings on gait adaptability. AR-based CMill gait adaptation training can improve short-term strategy selection, reaction time, and automated postural control during obstacle avoidance (Weerdesteyn et al., 2008). Gait speed is often referred to as the “sixth vital sign,” reflecting overall function and physiological changes, predicting patients’ recovery potential (Bishnoi et al., 2022). Yet, the lower importance and weaker correlation of ASL and AST suggest the immediate effects of treadmill interventions on interlimb differences. Harris et al. discovered that chronic stroke patients immediately improved their asymmetry after treadmill walking, reducing interlimb differences (Harris-Love et al., 2001). We need to consider the transfer and continuity of treadmill training effects on overground walking.

The ML algorithms proposed in previous studies demonstrated good classification performance (Al-Ramini et al., 2022). In our study, the SVM, DT, KNN, and MLP models exhibited different strengths in ACC (0.75–0.81), SEN (0.64–0.77), F1-score (0.56–0.69), precision (0.44–0.65), and ROC-AUC (0.6–0.7), but the overall performance was moderate. The initial concern may be related to feature selection. A review suggests that kinetic features yield higher accuracy as outputs (Alfayeed and Saini, 2021). However, for classifiers, features relevant to disease characteristics are also crucial for improving generalization (Li et al., 2019). Our study focused on GAD after stroke, so we retained all gait features and some general demographic features. This approach is similar to some ML models. Rodrigo et al. (2021) used demographics, clinical symptoms, and treatment data to predict the success of cognitive behavioral therapy for tinnitus with six algorithms with acceptable ACC (56.3–70.7%) and sensitivity (range 68.6–78.3%). Zhang et al. (2022) combined all kinematic gait parameters with walking tests to improve the diagnostic value for PD with an AUC of 0.924, but there was no training and testing data for their study.

Secondly, model performance is impacted by data imbalance. Consistent with previous medical ML studies (Cohen et al., 2006), our dataset has a significant class imbalance, with fewer samples in the stroke group. In our study, there were 30 stroke patients and 50 healthy controls, with a ratio of approximately 1.6, similar to the stroke-healthy ratio in the datasets of Hussain and Park (2021) and Luo et al. (2020). The two groups were matched for age, gender, height, and weight. When constructing stroke prediction models or hemiplegic gait classification using ML techniques, it is necessary to introduce appropriate algorithms (such as AdaCost) to mitigate the issue of low model accuracy caused by data imbalance. However, many datasets involve class imbalance issues, but the handling of this imbalance is not clearly defined, which may affect model performance. In our future work, we still need to further validate that AdaBoost is suitable in the real clinical setting. AdaCost ensemble learning algorithm considers the influence of minority class samples on overall classification accuracy, making it particularly suitable for small, imbalanced datasets (Jinzhu et al., 2009; Lakshmanarao et al., 2021). Compared to the well-known single classifier SVM in our study, AdaCost algorithm improved ACC by 10% (0.85), SEN by 5% (0.80), F1-score by 8% (0.77), and AUC by 7% (0.75), indicating this ensemble learning algorithm is effective and reliable. In developing a model to predict small ubiquitin-like modifier protein sites, Ye (2020) addressed data imbalance using the AdaCost algorithm and increased ACC by 0.25 and F1-score by 1.52. The high SEN of the AdaCost model indicates its strong capability to identify most individuals with GAD, ensuring patients receive the necessary interventions. The results of ACC and F1-score indicate the AdaCost model maintains a good balance between correctly identifying true cases and avoiding false positives. The AUC of 0.75 demonstrates that the model can discriminate cases, aligning with Chen’s model using CMill gait adaptation data to distinguish the FOG in PD (AUC = 0.755; Chen et al., 2021). While there is potential for further enhancement in the precision metric, it is crucial to evaluate performance metrics within the specific context of the medical application (Hicks et al., 2022). Early and accurate diagnosis of post-stroke GAD is critical for ensuring that patients receive timely and appropriate treatment. Therefore, the model should prioritize improving SEN over precision in this study.

Developing an aided diagnostic system based on ML algorithms can facilitate user-friendly interfaces suitable for clinical practice. This design is part of a computerized clinical decision support system (CDSS) which can enhance the complex decision-making process of clinicians (Sutton et al., 2020). Our study is the initial step toward this goal, showing some clinical feasibility. Kunhimangalam et al. (2014) developed a diagnostic system for peripheral neuropathy using fuzzy logic, taking symptoms and test results as input and achieving an accuracy of 93%. Google, IBM, and DeepMind have developed products used in CDSS, including tumor detection, automated tumor grading, and recurrence prediction through advanced pixel recognition and image classification algorithms (Sutton et al., 2020). Experts predict that most diagnostic imaging interpretations will be computer-performed or pre-processed in the future (Erickson, 2016). However, data quality, technical operability, maintenance, and financial considerations remain crucial. Currently, AI and CDSS technologies can complement clinical diagnosis. In our future studies, further data mining, extraction of appropriate features, continuous model training, and external validation are needed to improve diagnostic model performance and better serve clinical diagnosis.

### Limitations

The study still has limitations: 1. In this study, the participants were recruited from a single center with a small sample size. We did not split a specific validation dataset to verify the model, leading to a potential of model overfitting. Our future study should expand the sample size and design a larger, more diverse cohort study to validate the model’s efficacy and promote its generalizability. 2. We did not collect more gait kinematic and kinetic data, which may limit the accuracy of this model. In the future, integration of multimodal data will be necessary to enhance model performance, including joint angles, limb trajectories, ground reaction force, center of pressure, and electrophysiology. 3. The longitudinal data was unavailable to access the changes in gait adaptation over time. A prospective study should be conducted to explore the characteristics of GAD over time and assist in rehabilitation guidance. 4. We did not develop a user-friendly interface and computer-aided diagnosis system. AdaCost should be further trained and explored, integrating it into the workflow to facilitate ease of clinical decision-making for healthcare professionals.

## Conclusion

Stroke patients walk slower with shorter SL and more asymmetry of SL and ST. Their gait adaptability was decreased, particularly in obstacle avoidance and speed adaptation. Furthermore, faster gait speed and better performance in obstacle avoidance correlate with better functional mobility. The AdaCost classifier performs well in identifying individuals with GAD, facilitating clinical rehabilitation diagnostics and decision-making, particularly in obstacle avoidance and gait speed. This advances the future development of user-friendly interfaces and computer-aided diagnosis systems.

## Data availability statement

The raw data supporting the conclusions of this article will be made available by the authors, without undue reservation.

## Ethics statement

The studies involving humans were approved by this study was approved by the Ethics Committee of the First Affiliated Hospital of Zhejiang Chinese Medical University [registration number 2021-KL-187-02]. The studies were conducted in accordance with the local legislation and institutional requirements. The participants provided their written informed consent to participate in this study.

## Author contributions

HY: Conceptualization, Funding acquisition, Investigation, Methodology, Writing – original draft, Writing – review & editing. ZL: Data curation, Investigation, Writing – original draft. HZ: Data curation, Formal analysis, Writing – review & editing. KL: Data curation, Formal analysis, Software, Writing – original draft. YZ: Data curation, Methodology, Supervision, Writing – review & editing. ZG: Data curation, Methodology, Supervision, Writing – review & editing. YM: Data curation, Methodology, Supervision, Writing – review & editing. CS: Conceptualization, Supervision, Validation, Writing – review & editing.

## References

[ref1] Abdollahi M. Rashedi E. Jahangiri S. Kuber P. M. Azadeh-Fard N. Dombovy M. (2024). Fall risk assessment in stroke survivors: a machine learning model using detailed motion data from common clinical tests and motor-cognitive dual-tasking. Sensors 24:812. doi: 10.3390/s24030812, PMID: 38339529 PMC10857540

[ref2] Alfayeed S. M. Saini B. S. (2021). *Human gait analysis using machine learning: a review*. 2021 international conference on computational intelligence and knowledge economy (ICCIKE), Dubai, United Arab Emirates, pp. 550–554.

[ref3] Allen J. L. Kautz S. A. Neptune R. R. (2011). Step length asymmetry is representative of compensatory mechanisms used in post-stroke hemiparetic walking. Gait Posture 33, 538–543. doi: 10.1016/j.gaitpost.2011.01.004 21316240 PMC3085662

[ref4] Al-Ramini A. Hassan M. Fallahtafti F. Takallou M. A. Rahman H. Qolomany B.. (2022). Machine learning-based peripheral artery disease identification using laboratory-based gait data. Sensors (Basel) 22:7432. doi: 10.3390/s22197432, PMID: 36236533 PMC9572112

[ref5] Bak B. A. Jensen J. L. (2016). High dimensional classifiers in the imbalanced case. Comput. Stat. Data Anal. 98, 46–59. doi: 10.1016/j.csda.2015.12.009

[ref6] Baker R. Esquenazi A. Benedetti M. G. Desloovere K. (2016). Gait analysis: clinical facts. Eur. J. Phys. Rehabil. Med. 52, 560–574. 27618499

[ref7] Balasubramanian C. K. Bowden M. G. Neptune R. R. Kautz S. A. (2007). Relationship between step length asymmetry and walking performance in subjects with chronic hemiparesis. Arch. Phys. Med. Rehabil. 88, 43–49. doi: 10.1016/j.apmr.2006.10.004 17207674

[ref8] Balasubramanian C. K. Clark D. J. Fox E. J. (2014). Walking adaptability after a stroke and its assessment in clinical settings. Stroke Res. Treat. 2014:591013. doi: 10.1155/2014/591013, PMID: 25254140 PMC4164852

[ref9] Bishnoi A. Lee R. Hu Y. Mahoney J. R. Hernandez M. E. (2022). Effect of treadmill training interventions on spatiotemporal gait parameters in older adults with neurological disorders: systematic review and Meta-analysis of randomized controlled trials. Int. J. Environ. Res. Public Health 19:2824. doi: 10.3390/ijerph19052824, PMID: 35270516 PMC8909968

[ref10] Broström E. W. Esbjörnsson A. C. von Heideken J. Iversen M. D. (2012). Gait deviations in individuals with inflammatory joint diseases and osteoarthritis and the usage of three-dimensional gait analysis. Best Pract. Res. Clin. Rheumatol. 26, 409–422. doi: 10.1016/j.berh.2012.05.007, PMID: 22867935

[ref11] Bush K. Cowan N. Katz D. E. Gishen P. (1992). The natural history of sciatica associated with disc pathology. A prospective study with clinical and independent radiologic follow-up. Spine (Phila Pa 1976) 17, 1205–1212. doi: 10.1097/00007632-199210000-00013 1440010

[ref12] Chen Z. Y. Yan H. J. Qi L. Zhen Q. X. Liu C. Wang P.. (2021). C-gait for detecting freezing of gait in the early to middle stages of Parkinson's disease: a model prediction study. Front. Hum. Neurosci. 15:621977. doi: 10.3389/fnhum.2021.621977, PMID: 33828470 PMC8019899

[ref13] Cohen G. Hilario M. Sax H. Hugonnet S. Geissbuhler A. (2006). Learning from imbalanced data in surveillance of nosocomial infection. Artif. Intell. Med. 37, 7–18. doi: 10.1016/j.artmed.2005.03.002, PMID: 16233974

[ref14] Cui C. Bian G. B. Hou Z. G. Zhao J. Su G. Zhou H.. (2018). Simultaneous recognition and assessment of post-stroke Hemiparetic gait by fusing kinematic, kinetic, and electrophysiological data. IEEE Trans. Neural Syst. Rehabil. Eng. 26, 856–864. doi: 10.1109/TNSRE.2018.2811415, PMID: 29641390

[ref15] Dommershuijsen L. J. Isik B. M. Darweesh S. K. L. van der Geest J. N. Ikram M. K. Ikram M. A. (2020). Unraveling the association between gait and mortality-one step at a time. J. Gerontol. A Biol. Sci. Med. Sci. 75, 1184–1190. doi: 10.1093/gerona/glz282, PMID: 31807749 PMC7243583

[ref16] Erickson B. J. (2016). Machine intelligence in medical imaging. Leesburg: Society for Imaging Informatics, SIIM.

[ref17] Ferrarello F. Bianchi V. A. Baccini M. Rubbieri G. Mossello E. Cavallini M. C.. (2013). Tools for observational gait analysis in patients with stroke: a systematic review. Phys. Ther. 93, 1673–1685. doi: 10.2522/ptj.20120344 23813091

[ref18] Fraiwan L. Hassanin O. (2021). Computer-aided identification of degenerative neuromuscular diseases based on gait dynamics and ensemble decision tree classifiers. PLoS One 16:e0252380. doi: 10.1371/journal.pone.0252380, PMID: 34086723 PMC8177554

[ref19] Geerse D. J. Roerdink M. Marinus J. van Hilten J. J. (2021). Assessing walking adaptability in stroke patients. Disabil. Rehabil. 43, 3242–3250. doi: 10.1080/09638288.2020.1731852 32186408

[ref20] Guzelbulut C. Suzuki K. Shimono S. (2022). Singular value decomposition-based gait characterization. Heliyon 8:e12006. doi: 10.1016/j.heliyon.2022.e12006, PMID: 36478804 PMC9720564

[ref21] Harris E. J. Khoo I. H. Demircan E. (2022). A survey of human gait-based artificial intelligence applications. Front. Robot. AI 8:749274. doi: 10.3389/frobt.2021.749274, PMID: 35047564 PMC8762057

[ref22] Harris-Love M. L. Forrester L. W. Macko R. F. Silver K. H. Smith G. V. (2001). Hemiparetic gait parameters in overground versus treadmill walking. Neurorehabil. Neural Repair 15, 105–112. doi: 10.1177/154596830101500204, PMID: 11811252

[ref23] Hatwell J. Gaber M. M. Atif Azad R. M. (2020). Ada-WHIPS: explaining AdaBoost classification with applications in the health sciences. BMC Med. Inform. Decis. Mak. 20:250. Published 2020 Oct 2. doi: 10.1186/s12911-020-01201-2, PMID: 33008388 PMC7531148

[ref24] Hechifa A. Lakehal A. Nanfak A. Saidi L. Labiod C. Kelaiaia R.. (2024). Improved intelligent methods for power transformer fault diagnosis based on tree ensemble learning and multiple feature vector analysis. Electr. Eng. 106, 2575–2594. doi: 10.1007/s00202-023-02084-y

[ref25] Hicks S. A. Strümke I. Thambawita V. Hammou M. Riegler M. A. Halvorsen P.. (2022). On evaluation metrics for medical applications of artificial intelligence. Sci. Rep. 12:5979. Published 2022 Apr 8. doi: 10.1038/s41598-022-09954-8, PMID: 35395867 PMC8993826

[ref26] Hollands K. L. Pelton T. Wimperis A. Whitham D. Jowett S. Sackley C.. (2013). Visual cue training to improve walking and turning after stroke: a study protocol for a multi-Centre, single blind randomised pilot trial. Trials 14:276. doi: 10.1186/1745-6215-14-276, PMID: 24004882 PMC3846668

[ref27] Hussain I. Park S. J. (2021). Prediction of myoelectric biomarkers in post-stroke gait. Sensors (Basel) 21:5334. doi: 10.3390/s21165334, PMID: 34450776 PMC8399186

[ref28] Jinzhu Y. Yang L. Wei L. Dazhe Z. (2009). *A three-dimensional method for detection of pulmonary nodule*, pp. 1–4.

[ref29] Kunhimangalam R. Ovallath S. Joseph P. K. (2014). A clinical decision support system with an integrated EMR for diagnosis of peripheral neuropathy. J. Med. Syst. 38:38. doi: 10.1007/s10916-014-0038-9, PMID: 24692180

[ref30] Lakshmanarao A. Srisaila A. Kiran T. S. R. (2021). *Heart disease prediction using feature selection and ensemble learning techniques*. In: Proceedings of the 2021 third international conference on intelligent communication technologies and virtual Mobile networks (ICICV), Tirunelveli, India, 4–6 February, pp. 994–998.

[ref31] Lau H. Y. Tong K. Y. Zhu H. (2009). Support vector machine for classification of walking conditions of persons after stroke with dropped foot. Hum. Mov. Sci. 28, 504–514. doi: 10.1016/j.humov.2008.12.003, PMID: 19428134

[ref32] Li M. Tian S. Sun L. Chen X. (2019). Gait analysis for post-stroke Hemiparetic patient by multi-features fusion method. Sensors (Basel) 19:1737. doi: 10.3390/s19071737, PMID: 30978981 PMC6479843

[ref33] Lu H. Gao H. Ye M. Wang X. (2021). A hybrid ensemble algorithm combining AdaBoost and genetic algorithm for Cancer classification with gene expression data. IEEE/ACM Trans. Comput. Biol. Bioinform. 18, 863–870. doi: 10.1109/TCBB.2019.2952102, PMID: 31722484

[ref34] Luo G. Zhu Y. Wang R. Tong Y. Lu W. Wang H. (2020). Random forest-based classsification and analysis of hemiplegia gait using low-cost depth cameras. Med. Biol. Eng. Comput. 58, 373–382. doi: 10.1007/s11517-019-02079-7, PMID: 31853775

[ref35] Mengqi Z. Hehua Z. Wang X. Cheng P. (2020). Study on CART-based ensemble learning algorithms for predicting TBM tunneling parameters and classing surrounding rockmasses. Chin. J. Rock Mech. Eng. 39, 1860–1871. doi: 10.13722/j.cnki.jrme.2019.0924

[ref36] Nadeau S. Betschart M. Bethoux F. (2013). Gait analysis for poststroke rehabilitation: the relevance of biomechanical analysis and the impact of gait speed. Phys. Med. Rehabil. Clin. N. Am. 24, 265–276. doi: 10.1016/j.pmr.2012.11.007 23598262

[ref37] Park S. B. Hwang S. Zhang B. T. (2003). *Mining the risk types of human papillomavirus (HPV) by Ada cost, pp. 403–412*.

[ref38] Renedo D. Acosta J. N. Leasure A. C. Sharma R. Krumholz H. M. De Havenon A.. (2024). Burden of ischemic and hemorrhagic stroke across the US from 1990 to 2019. JAMA Neurol. 81, 394–404. doi: 10.1001/jamaneurol.2024.0190, PMID: 38436973 PMC10913004

[ref39] Rodrigo H. Beukes E. W. Andersson G. Manchaiah V. (2021). Exploratory data mining techniques (decision tree models) for examining the impact of internet-based cognitive behavioral therapy for tinnitus: machine learning approach. J. Med. Internet Res. 23:e28999. doi: 10.2196/28999, PMID: 34726612 PMC8596228

[ref40] Sang D. C. Lu L. P. Shao C. X. Liu H. R. Zhao Z. (2013). 3D gait analysis for old hemiplegic patients. Chin. J. Rehabil. Theory Pract. 19, 860–862. doi: 10.3969/j.issn.1006-9771.2013.09.017

[ref41] Soni S. Lamontagne A. (2021). Characterization of speed adaptation while walking on an omnidirectional treadmill. J. Neuroeng. Rehabil. 18:40. doi: 10.1186/s12984-02000787y 33618692 PMC7898772

[ref42] Sutton R. T. Pincock D. Baumgart D. C. Sadowski D. C. Fedorak R. N. Kroeker K. I. (2020). An overview of clinical decision support systems: benefits, risks, and strategies for success. NPJ Digit Med. 3:17. doi: 10.1038/s41746-020-0221-y, PMID: 32047862 PMC7005290

[ref43] Tuijtelaars J. Brehm M. A. Nollet F. Roerdink M. (2022). Validity and reproducibility of C-mill walking-adaptability assessment in polio survivors. Gait Posture 96, 314–321. doi: 10.1016/j.gaitpost.2022.06.008, PMID: 35772347

[ref44] Tuijtelaars J. Roerdink M. Raijmakers B. Nollet F. Brehm M. A. (2021). Polio survivors have poorer walking adaptability than healthy individuals. Gait Posture 87, 143–148. doi: 10.1016/j.gaitpost.2021.04.031, PMID: 33915437

[ref45] Van Ooijen M. W. Heeren A. Smulders K. Geurts A. C. Janssen T. W. Beek P. J.. (2015). Improved gait adjustments after gait adaptability training are associated with reduced attentional demands in persons with stroke. Exp. Brain Res. 233, 1007–1018. doi: 10.1007/s00221-014-4175-7, PMID: 25537466

[ref46] Van Swigchem R. Roerdink M. Weerdesteyn V. Geurts A. C. Daffertshofer A. (2014). The capacity to restore steady gait after a step modification is reduced in people with poststroke foot drop using an ankle-foot orthosis. Phys. Ther. 94, 654–663. doi: 10.2522/ptj.20130108, PMID: 24557646

[ref47] Weerdesteyn V. Nienhuis B. Duysens J. (2008). Exercise training can improve spatial characteristics of time-critical obstacle avoidance in elderly people. Hum. Mov. Sci. 27, 738–748. doi: 10.1016/j.humov.2008.03.003, PMID: 18524403

[ref48] Yang H. Gao Z. Zhou Y. Liao Z. Song C. Mao Y. (2024). Effects of gait adaptation training on augmented reality treadmill for patients with stroke in community ambulation. Int. J. Qual. Health Care 36:mzae008. doi: 10.1093/intqhc/mzae008, PMID: 38334696

[ref49] Ye S. (2020). *Prediction of Protein SUMO Modification Sites Based on Cost-Sensitive Learning*. East China Normal University, MA thesis.

[ref50] Ye Q. Xia Y. Yao Z. (2018). Classification of gait patterns in patients with neurodegenerative disease using adaptive neuro-fuzzy inference system. Comput. Math. Methods Med. 2018, 1–8. doi: 10.1155/2018/9831252, PMID: 30363986 PMC6186329

[ref51] Yin Q. Y. Zhang J. S. Zhang C. X. Liu S. C. (2013). An empirical study on the performance of cost-sensitive boosting algorithms with different levels of class imbalance. Math. Probl. Eng. 2013:761814. doi: 10.1155/2013/761814

[ref52] Zhang X. Fan W. Yu H. Li L. Chen Z. Guan Q. (2022). Single-and dual-task gait performance and their diagnostic value in early-stage Parkinson's disease. Front. Neurol. 13:974985. doi: 10.3389/fneur.2022.974985, PMID: 36313494 PMC9615249

[ref53] Zhong L. C. Wei H. Z. Dong X. Peng X. J. Zheng J. J. (2021). Advance in gait adaptability training for rehabilitation of stroke (review). Chin. J. Rehabil. Theory Pract. 27, 54–59. doi: 10.3969/j.issn.1006-9771.2021.01.008

