# Supplementary material for: Machine learning-based gait adaptation dysfunction identification using CMill-based gait data
Source: Front Neurorobot. 2024 Jul 29;18:1421401. doi: 10.3389/fnbot.2024.1421401 (PMC11317473; doi:10.3389/fnbot.2024.1421401)
Supplement: Supplementary file 1 [file Supplementary_material.docx]

**Supplementary materials**

**Supplementary Figure S1** CMill VR^+^ Treadmill


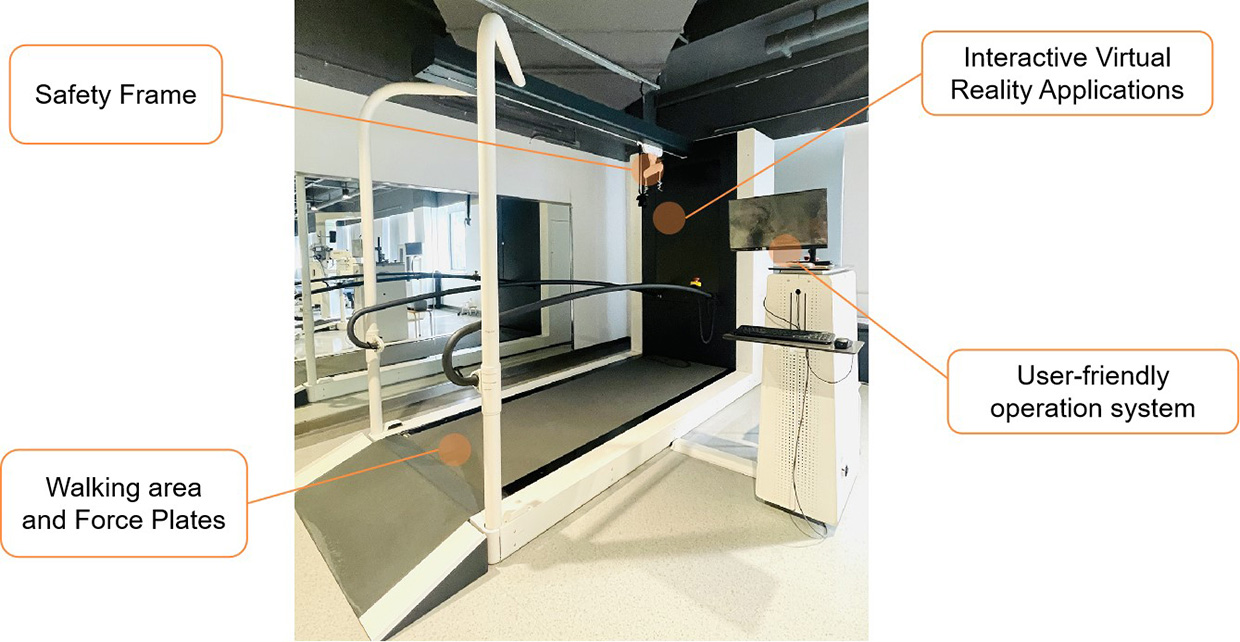


CMill VR+ treadmill consists of a screen with an interactive virtual reality application, a walking area with built-in force plates, a safety frame with safety harnesses, and a computer system for user-friendly operation.

**Supplementary Table S1** Definition and Explanation of the Composite Dataset

| **Data acquisition** | **Variables** | **Definition and explanation** | **Unit** |
| --- | --- | --- | --- |
| **Clinical baseline characteristics** | | | |
| Interview and measured by CMill | Age | Recorded through interview | y |
|  | Gender | Recorded through interview. Male are marked as 1 and female are marked as 0. | - |
|  | Height | Recorded before CMill walking | m |
|  | Weight | Recorded by CMill | kg |
|  | Onset of stroke | Recorded through interview | d |
|  | Affected side | Recorded through interview. Left hemiplegia is marked as 1, right is marked as 2 and healthy group are marked as 0. | - |
|  | FAC | Measured by a physician | level |
|  | ADL | Measured by a physician | score |
| **Gait kinematics (gait spatiotemporal parameters)** | | | |
| Raw data was collected by CMill. The variables were calculated and reported by CMill. | Gait speed | The distance walked by the participant in a unit of time. In this study, it was measured at the belt speed. | km/h |
|  | SL | The absolute displacement from one heel strike to the opposite heel strike within one gait cycle. | m |
|  | Step width | The lateral distance between the midpoints of the participant's feet within one gait cycle, averaged across each gait cycle during the entire test process to represent gait stability | m |
|  | ST | The duration during which one foot makes contact with and bears weight on the ground while the other foot is in the swing phase within one gait cycle. | s |
|  | ASL | longer SL / shorter SL | - |
|  | AST | longer ST / shorter ST | - |
| **Gait adaptability (gait adaptation task)** | | | |
| CMill calculated the success rate of task in a unified standard (0-100%). | Target stepping | Step on the target object. If you step on it successfully, the target object will be displayed in green. If you fail to step on it, it will be displayed in red. Success rate = number of successful targets/total targets*100% | % |
|  | Slalom walking | Walk along the curve. If any foot leaves the curve, an error message will be displayed. Success rate = number of steps in the curve / total number of steps * 100% | % |
|  | Obstacle avoidance | Both feet need to cross the obstacles. When completed, the footprints will be displayed in green. Success rate = number of successfully crossed obstacles / total number of obstacles * 100% | % |
|  | Speed adaptation | Both feet must stay in the changing target area. When any foot leaves, the footprint turns red. Success rate = number of steps in the target area / total number of steps * 100% | % |

SL, step length; ST, single stance time; ASL, asymmetry of step length; AST, asymmetry of single stance time

**Supplementary Table S2** The different machine learning algorithms

| SVM | The core concept of SVM is to create a hyperplane that serves as the decision boundary, maximizing the margin between samples of different classes. During training, we utilize the Radial Basis Function (RBF) kernel. |
| --- | --- |
| DT | Decision Trees (DT) are a collection of if-then rules and can also be considered as a conditional probability distribution defined over feature space and class space. During training, we use algorithms such as ID3, C4.5, and C5.0 to construct the trees. |
| MLP | It is a type of feedforward neural network. During training, the network adjusts its weights using the backpropagation algorithm to minimize the error between predicted values and actual values. |
| KNN | The core idea of the KNN algorithm is to compute the distance between the test sample and all training samples, selecting the K samples with the smallest distances. The test sample's class is then determined based on the class labels of these K nearest neighbors, typically through majority voting. |
| AdaCost | AdaCost is an algorithm that introduces a cost-sensitive mechanism to the sample weight update strategy of AdaBoost. It significantly increases the weights of misclassified samples with high costs while moderately decreasing the weights of correctly classified samples with high costs. We select decision trees generated by the CART algorithm as the base learners for the AdaCost classification mode. |

SVM, Support Vector Machine; DT, Decision Tree; MLP, Multi-Layer Perceptron; KNN, K-Nearest Neighbors; AdaCost, Adaptive Cost-Sensitive algorithm; AdaBoost, Adaptive Boosting algorithm

**Supplementary Table S3** Confusion matrix

| Actual results | Predicted results | |
| --- | --- | --- |
|  | Positive | Negative |
| Positive (Actual) | TP | FN |
| Negative (Actual) | FP | TN |

TP, true positive; FP, false positive; FN, false negative; TN, true negative

**Supplementary Table S4** Performance metrics on the confusion matrix

| **Performance Metrics** | **Calculation** |
| --- | --- |
| ACC | (TP+NT)/(TP+TN+FP+FN) |
| Precision | TP/(TP+FP) |
| SEN | TP/(TP+FN) |
| F1-score | 2*TP/(2*TP+FP+FN) |

ACC, accuracy; SEN, sensitivity; TP, true positive; FP, false positive; FN, false negative; TN, true negative
